# Peritumoral Imaging Manifestations on Gd-EOB-DTPA-Enhanced MRI for Preoperative Prediction of Microvascular Invasion in Hepatocellular Carcinoma: A Systematic Review and Meta-Analysis

**DOI:** 10.3389/fonc.2022.907076

**Published:** 2022-06-24

**Authors:** Ying Wu, Meilin Zhu, Yiming Liu, Xinyue Cao, Guojin Zhang, Longlin Yin

**Affiliations:** ^1^Department of Radiology, Sichuan Provincial People’s Hospital, University of Electronic Science and Technology of China, Chengdu, China; ^2^Department of Radiology, Affiliated Hospital of North Sichuan Medical College, Nanchong, China; ^3^Department of Radiology, Affiliated Hospital of Southwest Medical University, Luzhou, China

**Keywords:** Gd-EOB-DTPA-enhanced MRI, microvascular invasion, hepatocellular carcinoma, peritumoral enhancement, peritumoral hypointensity, meta-analysis

## Abstract

**Purpose:**

The aim was to investigate the association between microvascular invasion (MVI) and the peritumoral imaging features of gadolinium ethoxybenzyl DTPA-enhanced magnetic resonance imaging (Gd-EOB-DTPA-enhanced MRI) in hepatocellular carcinoma (HCC).

**Methods:**

Up until Feb 24, 2022, the PubMed, Embase, and Cochrane Library databases were carefully searched for relevant material. The software packages utilized for this meta-analysis were Review Manager 5.4.1, Meta-DiSc 1.4, and Stata16.0. Summary results are presented as sensitivity (SEN), specificity (SPE), diagnostic odds ratios (DORs), area under the receiver operating characteristic curve (AUC), and 95% confidence interval (CI). The sources of heterogeneity were investigated using subgroup analysis.

**Results:**

An aggregate of nineteen articles were remembered for this meta-analysis: peritumoral enhancement on the arterial phase (AP) was described in 13 of these studies and peritumoral hypointensity on the hepatobiliary phase (HBP) in all 19 studies. The SEN, SPE, DOR, and AUC of the 13 investigations on peritumoral enhancement on AP were 0.59 (95% CI, 0.41−0.58), 0.80 (95% CI, 0.75−0.85), 4 (95% CI, 3−6), and 0.73 (95% CI, 0.69−0.77), respectively. The SEN, SPE, DOR, and AUC of 19 studies on peritumoral hypointensity on HBP were 0.55 (95% CI, 0.45−0.64), 0.87 (95% CI, 0.81−0.91), 8 (95% CI, 5−12), and 0.80 (95% CI, 0.76−0.83), respectively. The subgroup analysis of two imaging features identified ten and seven potential factors for heterogeneity, respectively.

**Conclusion:**

The results of peritumoral enhancement on the AP and peritumoral hypointensity on HBP showed high SPE but low SEN. This indicates that the peritumoral imaging features on Gd-EOB-DTPA-enhanced MRI can be used as a noninvasive, excluded diagnosis for predicting hepatic MVI in HCC preoperatively. Moreover, the results of this analysis should be updated when additional data become available. Additionally, in the future, how to improve its SEN will be a new research direction.

## 1 Introduction

Hepatocellular carcinoma (HCC) is considered the global third highest cause of cancer mortality, ranking second among men (1). However, recurrence is common after surgical treatment. In addition, 5-year recurrence rates reach 70% after surgical resection and 35% after liver transplantation (2). In addition, microvascular invasion (MVI) has been identified as a possible predictor of early recurrence of HCC (3). MVI is considered to be the invasion of tumor cells into the vascular endothelium, which can only be seen under a microscope but not macroscopically. The presence of MVI suggests the aggressive behavior and poor survival outcome of HCC (4). A preoperative risk assessment of HCC patients by surgeons is of great importance. If radical hepatectomy is undertaken in patients at high risk for MVI, larger margins may be preferred; if liver transplantation is performed, the survival outcome of the patient is severely compromised (5). Histopathological examination is the gold standard for diagnosing MVI. However, histopathological examination is an invasive procedure that necessitates extensive sampling. Therefore, a preoperative, noninvasive test for detecting MVI would be extremely helpful in choosing the best treatment options for HCC patients (6). Both clinicians and patients benefit from preoperative noninvasive prediction of MVI.

Gadolinium ethoxybenzyl DTPA-enhanced (Gd-EOB-DTPA-enhanced) MRI uses a liver-specific, intracellular MRI contrast agent called Primovist or Eovist, which is distributed differently in various phases during the course of an MRI. In the arterial phase (AP), Primovist is distributed in vascular and extracellular regions. Gradually, it is distributed in bile ducts and hepatocytes in the hepatobiliary phase (HBP) (7). Gd-EOB-DTPA provides insight into hemodynamic changes in the liver and liver tumors. Gd-EOB-DTPA-enhanced MRI is not only helpful in the diagnosis of HCC but has also been widely applied to the preoperative evaluation and prognostic evaluation of HCC (8, 9). In addition, gadobenate dimeglumine (Gd-BOPTA) is a liver-specific contrast agent. The T1 relaxivity at 1.5T for Primovist and Gd-BOPTA is 6.5−7.3 and 6−6.6, respectively (10). Moreover, the protein-binding capabilities of Gd-BOPTA are weaker than that of Gd-EOB-DTPA, and its uptake by hepatocytes is about one-tenth of the amount of Gd-EOB-DTPA, which might be related to the difference in the lipophilicity of the benzene ring in Gd-BOPTA and the EOB group in Gd-EOB-DTPA (10–12).

Recently, some studies have focused on the imaging findings of HCC tumors themselves to predict the relationship of MVI (13–15). However, based on the altered hemodynamics, peritumoral tissue is the first tissue that is affected by MVI. It is worthy to explore whether peritumoral tissue can directly reflect the relationship between tumor and MVI. Moreover, a high-quality meta-analysis showed that peritumoral enhancement on AP and peritumoral hypointensity on HBP were associated with MVI but with poor diagnostic accuracy (16). However, the number of included literatures in the publication was small, with only four articles about peritumoral hypointensity on HBP, and 2 studies used CT to assess peritumoral enhancement (16). Moreover, the research did not use Primovist as a contrast agent. However, Ahn SJ et al. and Ahn, S Y. et al. found that peritumoral enhancement on AP and peritumoral hypointensity on HBP did not show a statistically significant association with MVI (*P* > 0.05) (17, 18). In addition, the reported SEN and SPE of peritumoral hypointensity on HBP varied widely—0.38−0.81 and 0.56−0.97, respectively (8, 9, 17–33). Yet, as the peritumoral microenvironment has received more attention in recent years, papers on the link between peritumoral imaging and MVI have been updated. Therefore, it is critical to determine the actual accuracy of the two imaging features for predicting the presence of MVI in HCC. As a result, the value of assessing the association between peritumoral imaging features and MVI by taking advantage of Gd-EOB-DTPA-enhanced MRI remains to be investigated.

On the whole, the predictive value of peritumoral enhancement on AP and peritumoral hypointensity on HBP on Gd-EOB-DTPA-enhanced MRI for MVI in HCC patients remains controversial. Furthermore, there has been no systematic evaluation of the diagnostic significance of these imaging findings of preoperative Gd-EOB-DTPA-enhanced MRI for MVI. Hence, this research was performed to determine the diagnostic performance of these features for MVI in HCC patients.

## 2 Methods

### 2.1 Literature Search Strategy

This study was conducted in accordance with the Preferred Reporting Items for Systematic Reviews and Meta-Analyses guidelines (34). Up until Feb 24, 2022, the PubMed, Embase, and Cochrane Library databases were carefully searched for relevant material by two researchers. Medical subject headings, free words, and their variations were employed for retrieval. Literature retrieval has no language restrictions. The full search strategy is described in the Supplementary material.

### 2.2 Inclusion and Exclusion Criteria

The criteria for selecting the subjects were as follows: 1) studies on preoperative MVI prediction with peritumoral tissue on disodium gadoxetate–enhanced MRI; 2) studies without treatment before curative hepatectomy; 3) histopathologically proven primary HCC; and 4) studies providing sufficient data to create a diagnostic 2 × 2 table. Further, the following circumstances would be excluded: 1) studieses that did not satisfy any of the aforementioned inclusion criteria; 2) reviews, letters, and reports; 3) studies for involving macrovascular invasion; and 4) studies for which we were unable to get the full text.

### 2.3 Quality Assessment and Data Extraction

This paper assessed the methodological quality of each study, applying the Quality Assessment of Diagnostic Accuracy Studies (QUADAS-2) tool (35). In addition, a comprehensive evaluation of the bias risk for each research was conducted, including patient selection, index test, reference standard, flow and timing, and applicability concerns. Meanwhile, two researchers independently extracted the data and cross-checked them to arrive at an agreement. In addition, the extracted data from each included study consisted of the first author, year of publication, region, lesion size, sample size of tumors and patients, single tumors or multiple, interval between imaging and surgery, magnetic field strengths, preoperative anti-tumor therapy, microvascular invasion, macrovascular invasion, and blindness to reference and index test. Moreover, the third researcher collated the extracted data as true positives, false positives, false negatives, and true negatives to form a 2 × 2 diagnostic table.

### 2.4 Definition of Peritumoral Enhancement and Peritumoral Hypointensity

Each study reached a consensus on the definition of peritumoral enhancement on AP and peritumoral hypointensity on HBP. Peritumoral enhancement on AP is defined as a polygonal-shaped or crescent-shaped enhancement outside the cancer edge during the AP, which becomes isointense to background hepatic parenchyma in the delayed phase (21). The definition of peritumoral hypointensity on HBP is considered as a flame-like or wedge-shaped hypointense region of hepatic parenchyma outside the edge of tumor during the HBP (23).

### 2.5 Statistical Analysis

Review Manager 5.4.1, Meta-DiSc 1.4, and Stata16.0 were used for data analysis and statistics. The evaluation indexes of diagnostic efficiency include SEN, SPE, positive likelihood ratio (PLR), negative likelihood ratio (NLR), diagnostic odds ratio (DOR), and 95% confidence interval (CI). Further, the diagnostic precision of peritumoral imaging features on Gd-EOB-DTPA-enhanced MRI for the prediction of MVI was analyzed using the area under the receiver operating characteristic curve (AUC). The Spearman correlation coefficient in Meta-DiSc1.4 was adopted to evaluate heterogeneity caused by the threshold effect. There was a significant threshold effect, as evidenced by a strong positive association (*P* < 0.05) (36). The heterogeneity of studies was determined by applying Cochran’s Q test and *I^2^* analysis and regarded as *P* < 0.1 or *I^2^* > 50% (37). In the case of notable heterogeneity, the random-effects coefficient binary regression model was utilized; otherwise, the fixed-effects coefficient binary regression model was employed (38). In addition, the causes of heterogeneity were investigated by subgroup analysis; the stability of this meta-analysis was estimated by sensitivity analysis, and the publication bias was detected by Deeks’ funnel plot asymmetry tests. If the slope coefficient was greater than zero, publication bias was suspected (*P* < 0.05) (39).

## 3 Results

### 3.1 Literature Search and Study Selection

**Figure 1:**
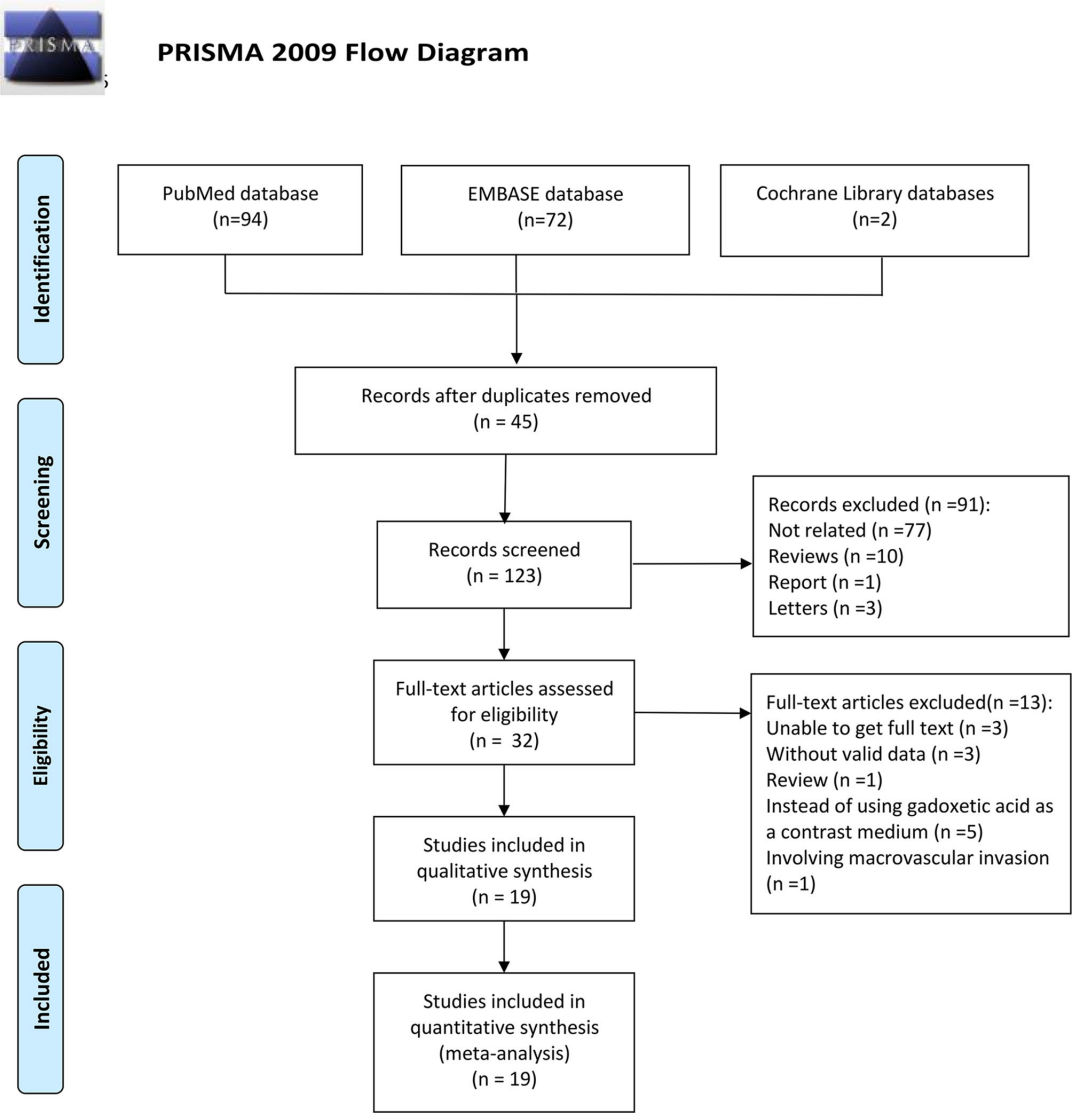
Flow diagram illustrating the search strategy.

Following the research approach, 168 publications were obtained *via* PubMed, Embase, and Cochrane Library databases. Forty-five articles were removed as duplicates (**Figure 1**). Moreover, 91 articles were eliminated after a review of titles and abstracts on the basis of the following reasons: publications were not related to the prediction of MVI or were reviews or report or letters, leaving 32 studies for further screening. After checking for the full text, a review was excluded, and 3 investigations were eliminated due to the unavailability of the full text, 3 for not having valid data, 5 for not using gadoxetic acid as a contrast medium, and one for involving macrovascular invasion. Finally, a total of nineteen articles were involved in this paper and their characteristics are listed in **Table 1**.

**Table 1:** Characteristics of the 19 included studies.

Study	Year	Region	Mean age (years)	Patients/Lesions (n)	Lesions	Lesions size	IBIS (days)	PEAP (n)		PHHBP (n)		MVI (n)	
								+	−	+	−	+	−
Ahn SJ. et al (17)	2019	South Korea	56.71	179 (179)	S	NR	≤30	64	115	61	118	68	111
Ahn SY. et al (18)	2015	South Korea	51.94	51 (78)	S/M	NR	≤63	10	68	4	74	18	60
Chen PP. et al (20)	2019	China	58	70 (77)	S/M	NR	≤14	15	62	20	57	27	50
Chen Y. et al (21)	2021	China	51.5	269 (269)	U	NR	≤14	73	196	105	164	111	158
Chong HH. et al (22)	2020	China	54.22	356 (356)	S	≤5 cm	≤30	74	282	54	302	90	266
Chou YC. et al (23)	2019	China	64.76	114 (114)	S	NR	U	27	87	34	80	39	75
Dong SY. et al (24)	2022	China	54.66	214 (214)	S	≤3 cm	≤30	79	135	75	139	49	165
Feng ST. et al (9)	2019	China	54.8	160 (160)	S/M	NR	≤30	44	116	48	112	62	98
Huang M. et al (8)	2018	China	52.2	60 (66)	S/M	NR	≤30	21	45	26	40	17	49
Kim KA. et al (19)	2012	South Korea	55	104 (104)	S/M	NR	≤30	NM	NM	26	78	60	44
Lee S. et al (25)	2020	South Korea	54	122 (122)	S/M	NR	≤30	NM	NM	21	101	21	101
Lu XY. et al (26)	2020	China	57.5	102 (102)	U	NR	≤30	NM	NM	26	76	31	71
Nishie A. et al (27)	2014	Japan	67	61 (61)	S/M	NR	≤30	NM	NM	25	36	25	36
Shin SK. et al (28)	2017	South Korea	57	126 (126)	S	≤5 cm	U	NM	NM	15	111	29	97
Wang LL. et al (29)	2021	China	54.22	113 (113)	S/M	NR	≤14	NM	NM	67	46	50	63
Yang L. et al (30)	2019	China	55.5	208 (208)	S/M	NR	≤30	67	141	30	178	53	155
Yang Y. et al (31)	2021	China	52.4	201 (201)	S	NR	≤30	111	90	82	119	111	90
Zhang K. et al (32)	2022	China	56.4	129 (129)	S	NR	≤30	49	80	43	86	36	93
Zhou M. et al (33)	2021	China	55	60 (62)	S/M	≤3 cm	≤30	14	48	12	50	19	43

IBIS, interval between imaging and surgery; PEAP, peritumoral enhancement on arterial phase; PHHBP, peritumoral hypointensity on hepatobiliary phase; MVI, microvascular invasion; +, positive; -, negative; S, single; M, multiple; U, unclear; NR, no restriction; NM, not mentioned.

### 3.2 Study Characteristics and Quality Assessment

A total of nineteen articles were included, and all studies examined peritumoral hypointensity on HBP, and 13 studies examined peritumoral enhancement on AP. Furthermore, all articles were retrospective studies. The studies were published between 2011 and 2022. Among these studies, 13 were from China, 5 from South Korea, and 1 from Japan. All 19 studies included 2,699 HCC patients with 2,741 tumors, of which 916 tumors were pathologically diagnosed as MVI-positive and 1,825 tumors as MVI-negative.

**Figure 2:**
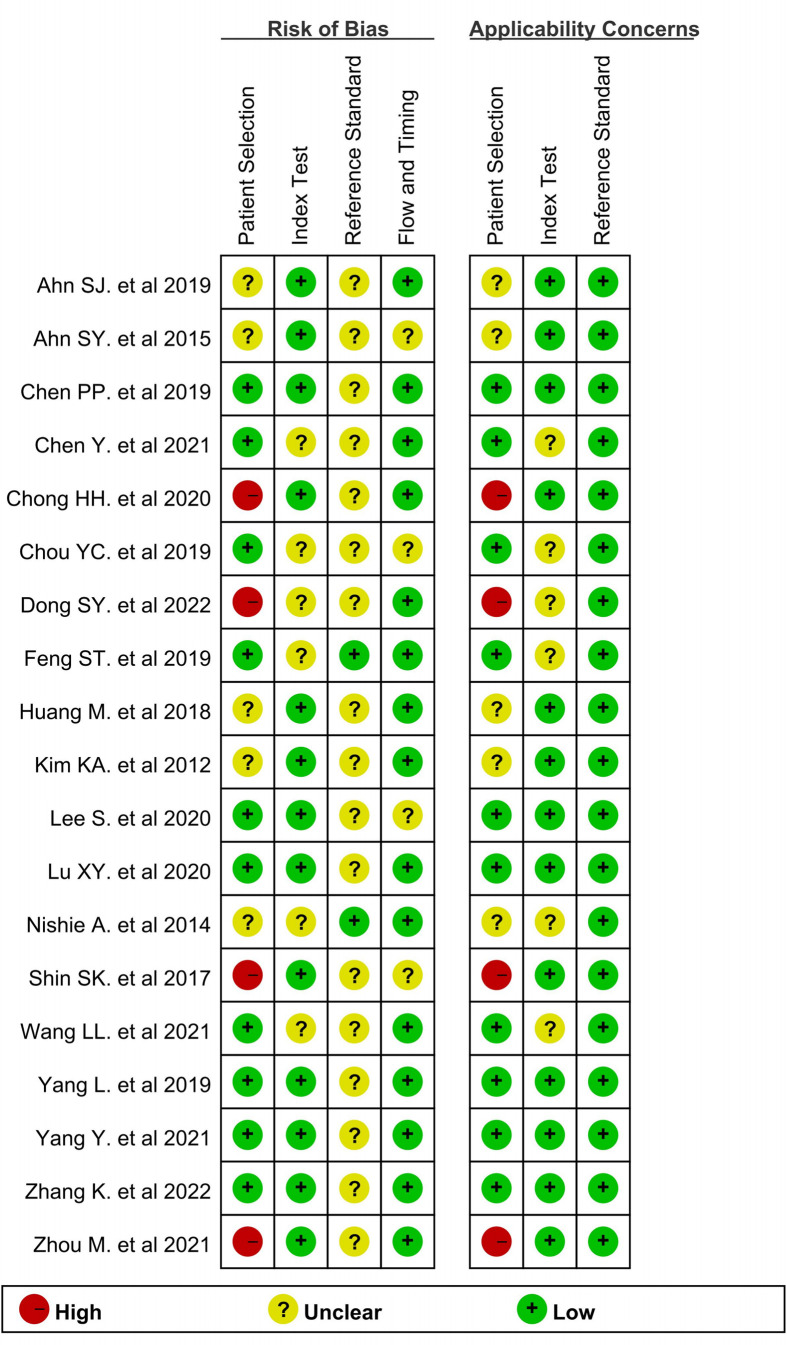
Methodological quality summary of all included studies by using Quality Assessment of Diagnostic Accuracy Studies.

**Figure 2** depicts the quality of the included investigations as assessed by QUADAS-2 guidelines. As it was not clear whether patients received other treatments before the operation in 2 studies (19, 28), the risk bias arising from patient selection in those studies was determined to be “unclear.” Due to the fact that the seven studies did not mention whether there was macrovascular invasion, we also marked it as an unclear risk of patient selection bias (8, 17–19, 24, 27, 28). Moreover, because the lesion size was limited in 4 studies (22, 24, 28, 33), the patient selection bias was considered as “high.” Six studies did not mention whether the radiologists were blinded to the pathology data (9, 21, 23, 24, 27, 29) and were therefore marked as unclear risk of index bias domain. The interval between imaging and surgery was unclear in 2 studies (23, 28); hence, the risk bias arising from flow and timing was determined to be “unclear.” All tumors were subjected to MRI examination and a histopathological test. Although most articles did not explicitly mention that “pathologists were blinded to the imaging data,” they did elaborate on the pathological findings. Accordingly, the risk bias arising from the reference standard was determined to be “unclear,” but in this research, we considered the applicability concerns of reference standard as “low concern.”

### 3.3 Imaging Methods

The characteristics of the imaging methods for the included studies are listed in **Table 2**. Ten studies reported MRI performed with a field strength of 3T, 4 studies used both 1.5T and 3T MRI systems, and 5 studies used 1.5T. In addition, ten articles used Siemens MR devices, while the rest used Philips/GE or two and three devices. Moreover, the scan acquisition time of the AP of 10 studies was performed at 20−35 s following the contrast injection. Three studies scanned AP seven seconds after the contrast media had arrived at the distal thoracic aorta and one when the contrast medium was visible at the level of the celiac trunk of the abdominal aorta. Additionally, the remaining five articles did not illustrate the scan acquisition time of AP. In all studies, the scan acquisition time for HBP was 20 min after the contrast injection. Moreover, the injection dose of Gd-EOB-DTPA was 0.025 mmol/kg body weight in 11 studies and 0.1 ml/kg in 5 studies, and one study injected the contrast in the dose of 0.2 ml/kg. One study used a bolus injection of 10 ml. In addition, one study did not specify the dose of contrast injection. The injection rate was 1 ml/s in 6 studies, 1.5 ml/s in 2 studies, and 1.0−1.5 ml/s in 2 studies. Aditionally, one article injected the contrast agent at a rate of 2 ml/s. The remaining studies did not mention the injection rate.

**Table 2:** Characteristics of imaging methods.

Study	MFS(T)	Scanners	Scan acquisition time		Doses of contrast agent	Injection flow rate
			AP	HBP		
Ahn SJ. et al (17)	1.5/3	GE/Siemens/Philips	Seven seconds after the contrast media had arrived at the distal thoracic aorta	20 min*	0.025 mmol/kg	1.5 ml/s
Ahn SY. et al (18)	1.5/3	GE/Siemens	Seven seconds after the contrast media had arrived at the distal thoracic aorta	20 min*	0.025 mmol/kg	1.5 ml/s
Chen PP. et al (20)	3	Philips	20 s*	20 min*	0.1 ml/kg	1.0−1.5 ml/s
Chen Y. et al (21)	3	Siemens	NM	20 min*	0.2 ml/kg	1 ml/s
Chong HH. et al (22)	1.5	Siemens	20−30 s*	20 min*	0.025 mmol/kg	NM
Chou YC. et al (23)	1.5	Siemens	When the contrast medium was visible at the level of the celiac trunk of the abdominal aorta.	20 min*	Bolus injection of 10 ml	1 ml/s
Dong SY. et al (24)	1.5	Siemens	20−30 s*	20 min*	0.025 mmol/kg	NM
Feng ST. et al (9)	3	Siemens	30–35 s*	20 min*	0.1 ml/kg	1 ml/s
Huang M. et al (8)	3	Siemens	NM	20 min*	NM	NM
Kim KA. et al (19)	3	Siemens	NM	10–20 min*	0.025 mmol/kg	2.0 ml/s
Lee S. et al (25)	1.5/3	Siemens/Philips	20–35 s*	20 min*	0.025 mmol/kg	1 ml/s
Lu XY. et al (26)	3	Philips	20 s*	10 and 20 min*	0.1 ml/kg	1.0–1.5 ml/s
Nishie A. et al (27)	1.5	Philips	NM	20 min*	0.1 ml/kg (total amount: 4.5–8 ml)	NM
Shin SK. et al (28)	3	Siemens	Seven seconds after the contrast media had arrived at the distal thoracic aorta	20 min*	0.025 mmol/kg	NM
Wang LL. et al (29)	3	Siemens	20−30 s*	20 min*	0.025 mmol/kg	1 ml/s
Yang L. et al (30)	1.5	Siemens	20–30 s*	20 min*	0.025 mmol/kg	NM
Yang Y. et al (31)	1.5/3	GE	20–35 s*	20 min*	0.025 mmol/kg	NM
Zhang K. et al (32)	3	Philips	NM	20 min*	0.025 mmol/kg	NM
Zhou M. et al (33)	3	Philips	25 s*	20 min*	0.1 ml/kg	1 ml/s

^*^This acquisition time is defined as after contrast media injection. MFS, magnetic field strength; AP, arterial phase; HBP, hepatobiliary phase; NM, no specific time point was mentioned.

### 3.4 Accuracy of Peritumoral Imaging Features of HCC for Predicting MVI

#### 3.4.1 Peritumoral Enhancement on AP

**Figure 3:**
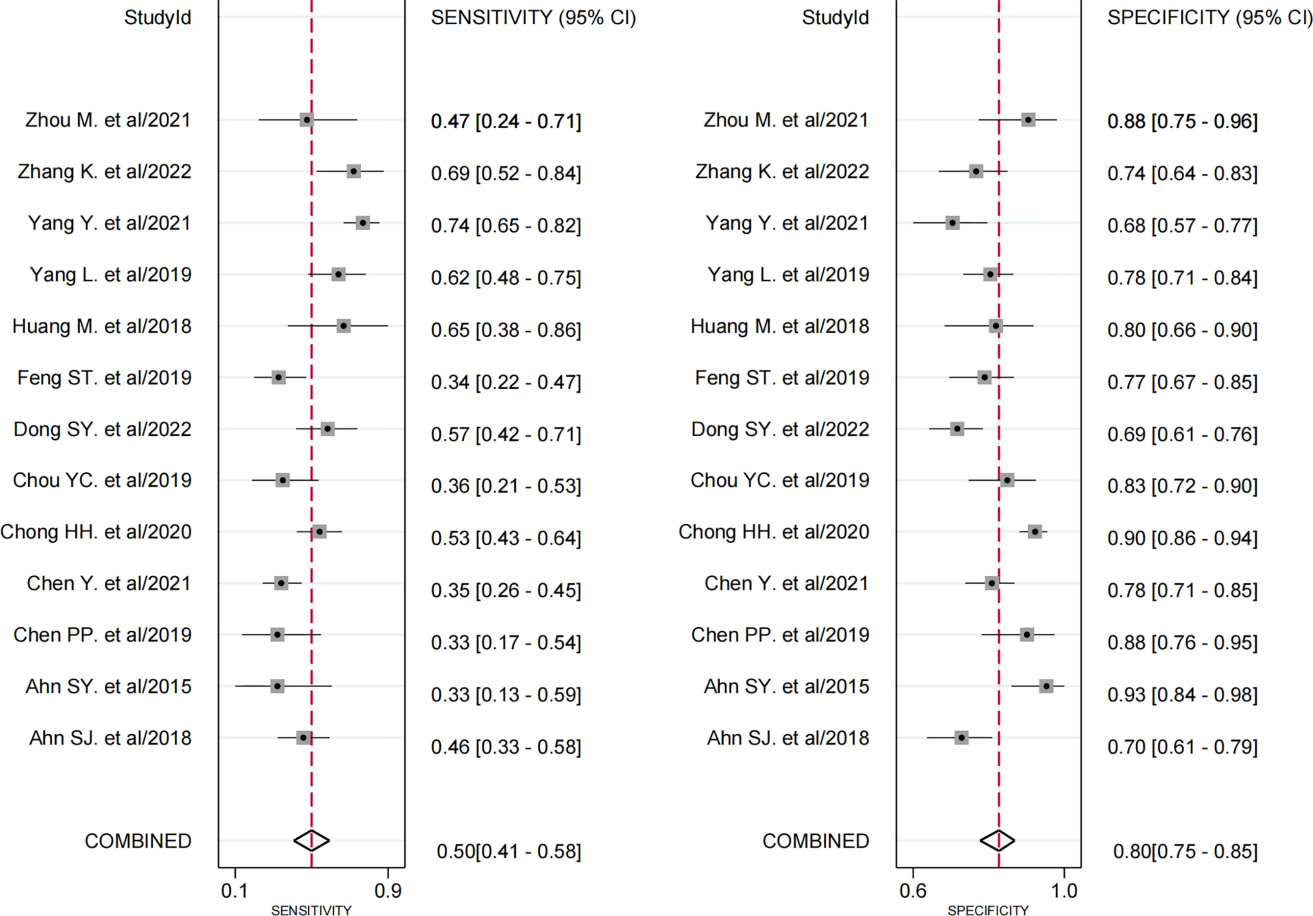
Forest plots demonstrate the pooled sensitivity and specificity of peritumoral enhancement on the arterial phase. The 95% CI are shown around point estimates and the pooled result.

**Figure 4:**
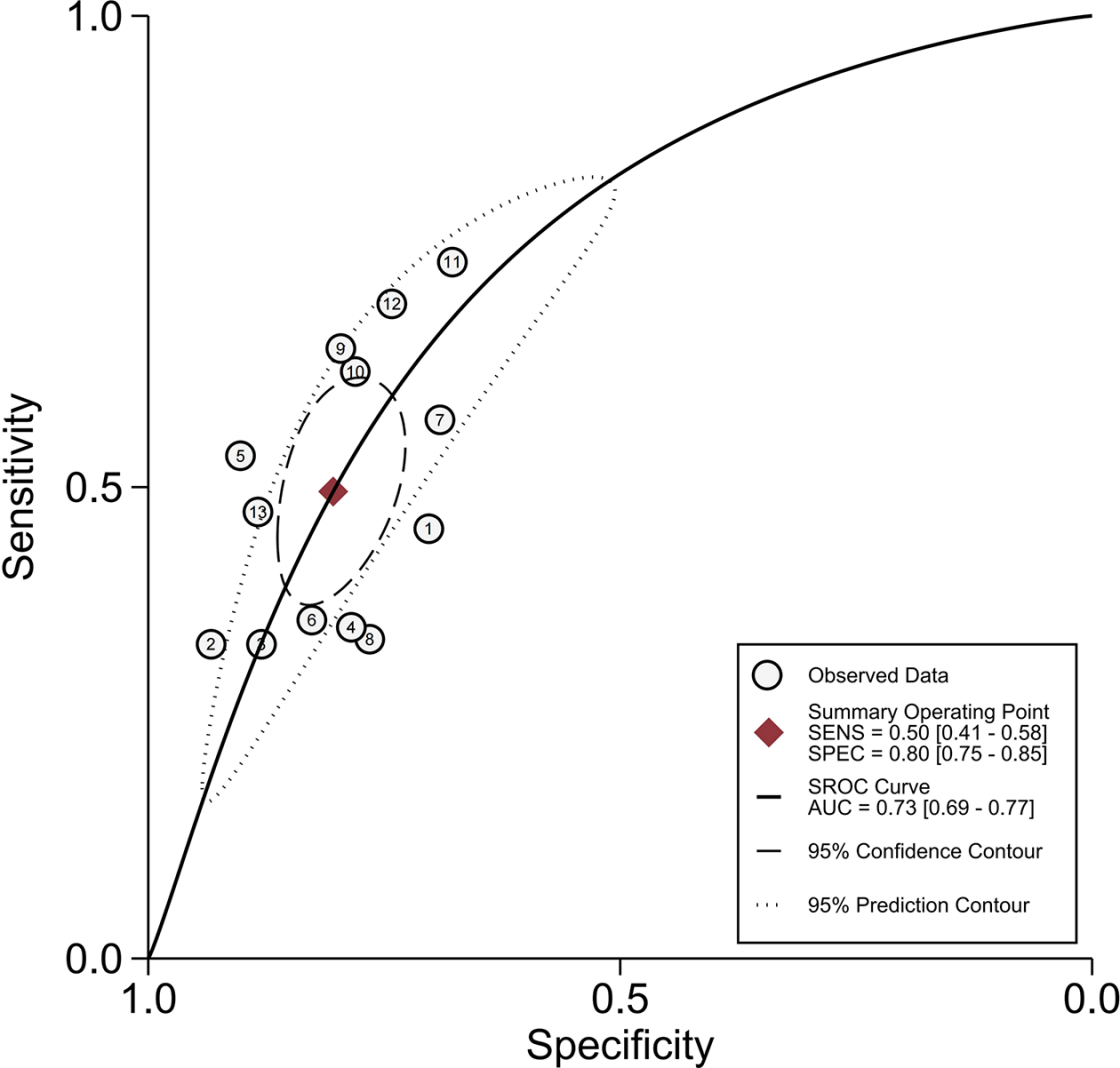
Summary receiver operating characteristic curves of peritumoral enhancement on the arterial phase.

Thirteen studies assessed the relationship between peritumoral enhancement on AP with Gd-EOB-DTPA-enhanced MRI and MVI (8, 9, 17, 18, 20–24, 30–33), including 2,071 HCC patients with 2,113 tumors. Of 2,113 tumors, 700 were pathologically diagnosed as MVI-positive (356 tumors with peritumoral enhancement on AP and 344 tumors without) and 1,413 as MVI-negative (292 tumors with peritumoral enhancement on AP and 1,121 tumors without). The Spearman correlation coefficient was 0.531 (*P* = 0.062), which indicated that threshold effect–derived heterogeneity was not present. The results of Cochran’s Q test and *I^2^* analysis (*P* < 0.001, *I^2^* = 95%) indicated that there was substantial heterogeneity. The pooled SEN was 0.50 (95% CI, 0.41−0.58), and the pooled SPE was 0.80 (95% CI, 0.75−0.85) (**Figure 3**). Moreover, the values of pooled PLR, NLR, and DOR were 2.5 (95% CI, 2.0−3.2), 0.63 (95% CI, 0.54−0.73), and 4 (95% CI, 3−6), respectively. In addition, the SROC curve was plotted (**Figure 4**), resulting in an AUC of 0.73 (95% CI, 0.69−0.77).

#### 3.4.2 Peritumoral Hypointensity on HBP

**Figure 5:**
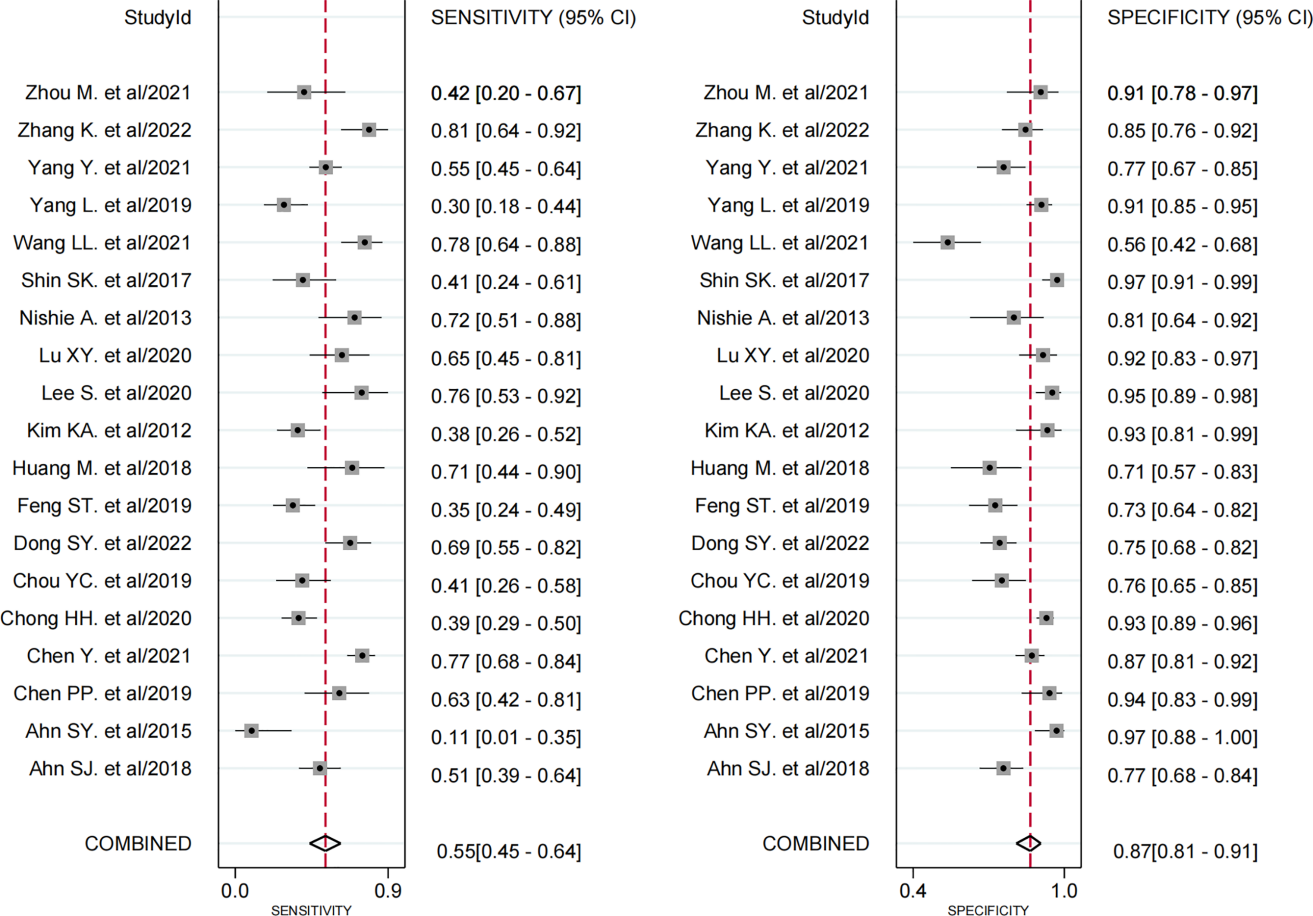
Forest plots demonstrate the pooled sensitivity and specificity of peritumoral hypointensity on the hepatobiliary phase. The 95% CI are shown around point estimates and the pooled result.

**Figure 6:**
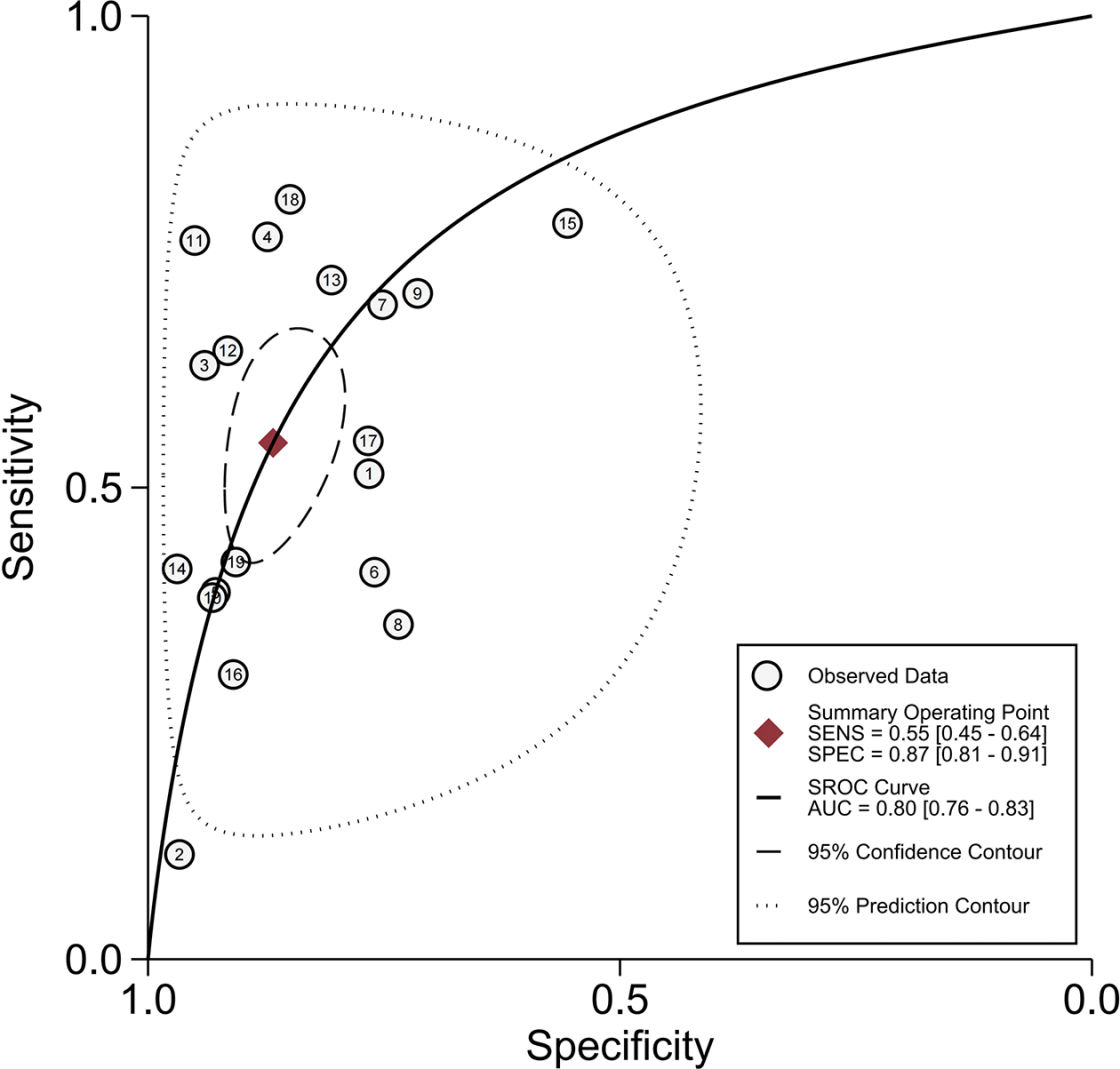
Summary receiver operating characteristic curves of peritumoral hypointensity on the hepatobiliary phase.

All 19 studies (8, 9, 17–33) provided the relevant data of peritumoral hypointensity on HBP to predict MVI in HCC with disodium gadoxetate–enhanced MRI, including 2,699 HCC patients with 2,741 tumors. Of 2,741 tumors, 916 were pathologically diagnosed as MVI-positive (500 tumors with peritumoral hypointensity on HBP and 416 tumors without) and 1,825 as MVI-negative (274 tumors with peritumoral hypointensity on HBP and 1,551 tumors without). Additionally, the Spearman correlation coefficient was 0.318 (*P* = 0.185), indicating the absence of threshold effect–derived heterogeneity. There was, however, significant heterogeneity among the included articles (*P* < 0.001, *I^2^* = 98%). The results of pooled SEN and SPE were 0.55 (95% CI, 0.45−0.64) and 0.87 (95% CI, 0.81−0.91), respectively (**Figure 5**). In addition, the pooled PLR, NLR, and DOR, separately, were 4.1 (95% CI, 3.0−5.7), 0.52 (95% CI, 0.43−0.63), and 8 (95% CI, 5−12). In addition, the AUC was 0.80 (95% CI, 0.76−0.83) (**Figure 6**).

### 3.5 Subgroup Analysis

The causes of pooled variability were investigated using subgroup analysis. Based on clinical experience and the classification of basic information from the included literature, subgroups were formed as follows: 1) region (China as “1,” others as “0”); 2) the mean age of included patients (≥55 years as “1,” <55 years as “0”); 3) magnetic field strength (only 3T as “1,” 1.5T or mixed as “0”); 4) MRI unit (Siemens as “1,” Philips/GE or mixed as “0”); 5) the lesion size of HCC (no restriction as “1,” ≤5 cm as “0”); 6) number of included tumors (≥100 as “1,” <100 as “0”); 7) only a single HCC (yes as “1,” multiple or mixed as “0”); 8) interval between imaging and surgery (≤30 days as “1,” >30 days as “0”); 9) without macrovascular invasion (yes as “1,” unclear as “0”); 10) blind to pathological outcomes (yes as “1,” unclear as “0”); and 11) blind to imaging diagnosis (yes as “1,” unclear as “0”).

**Tables 3** and **4** present the outcomes of the subgroup analysis. Except for blindness to the index test during the pathological test, the above ten covariates were major determinants in causing heterogeneity, according to the results of peritumoral enhancement on AP (*P* < 0.05). Additionally, in terms of peritumoral hypointensity on HBP, the findings demonstrated that the region, mean age of included patients, magnetic field strength, MRI unit, number of included tumors, and only a single HCC, as well as the interval between imaging and surgery, are significant sources of heterogeneity (*P* < 0.05).

**Table 3:** Subgroup analyses of peritumoral enhancement on arterial phase.

Variable	Subgroups	Studies (n)	Sensitivity	P1	Specificity	P2
Region	China	11	0.51 (0.42−0.60)	0.31	0.80 (0.75−0.85)	0.03
	Others	2	0.39 (0.18−0.59)		0.82 (0.71−0.94)	
Mean age (years)	≥55	6	0.49 (0.36−0.62)	0.96	0.80 (0.73−0.87)	0.00
	<55	7	0.50 (0.39−0.61)		0.80 (0.74−0.87)	
MFS (T)	3	6	0.45 (0.33−0.57)	0.57	0.81 (0.74−0.88)	0.00
	Others	7	0.53 (0.42−0.64)		0.80 (0.74−0.86)	
MRI unit	Siemens	7	0.48 (0.37−0.59)	0.78	0.80 (0.74−0.86)	0.00
	Others	6	0.52 (0.39−0.64)		0.81 (0.74−0.88)	
Lesion size	≤5 cm	3	0.53 (0.36−0.71)	0.76	0.84 (0.76−0.92)	0.03
	NR	10	0.48 (0.39−0.58)		0.79 (0.74−0.85)	
No. of tumors (n)	≥100	9	0.52 (0.43−0.61)	0.48	0.77 (0.72−0.82)	0.00
	<100	4	0.44 (0.27−0.61)		0.88 (0.82−0.94)	
Lesions	S	6	0.57 (0.46−0.67)	0.21	0.77 (0.70−0.84)	0.00
	Others	7	0.43 (0.32−0.53)		0.83 (0.77−0.89)	
IBIS (days)	≤30	11	0.52 (0.44−0.61)	0.14	0.79 (0.74−0.84)	0.00
	>30	2	0.34 (0.14−0.54)		0.88 (0.80−0.96)	
Macro VI	N	9	0.50 (0.40−0.60)	0.91	0.81(0.76−0.86)	0.01
	U	4	0.49 (0.33−0.65)		0.78 (0.69−0.87)	
BR	Y	9	0.55 (0.46−0.64)	0.13	0.82 (0.77−0.88)	0.02
	U	4	0.40 (0.28−0.52)		0.77 (0.68−0.86)	
BI	Y	1	0.34 (0.09−0.58)	0.45	0.77 (0.58−0.95)	0.16
	U	12	0.51 (0.43−0.59)		0.81 (0.76−0.86)	

Data in parentheses are 95% confidence interval (CI). MFS, magnetic field strength; IBIS, interval between imaging and surgery; Macro VI, macrovascular invasion, BR, blindness to reference; BI, blindness to index test; NR, no restrictions; S, single; M, multiple; U, unclear; N, no, Y, yes.

**Table 4:** Subgroup analyses of peritumoral hypointensity on hepatobiliary phase.

Variable	Subgroups	Studies (n)	Sensitivity	P1	Specificity	P2
Region	China	13	0.58 (0.47−0.69)	0.58	0.84 (0.78−0.90)	0.00
	Others	6	0.48 (0.31−0.64)		0.92 (0.87−0.97)	
Mean age (years)	≥55	10	0.52 (0.40−0.65)	0.48	0.89 (0.84−0.94)	0.03
	<55	9	0.57 (0.44−0.71)		0.84 (0.77−0.91)	
MFS (T)	3	10	0.60 (0.48−0.72)	0.59	0.87 (0.80−0.93)	0.01
	Others	9	0.49 (0.36−0.62)		0.87 (0.80−0.93)	
MRI unit	Siemens	10	0.52 (0.40−0.65)	0.48	0.84 (0.78−0.91)	0.00
	Others	9	0.58 (0.44−0.71)		0.89 (0.84−0.95)	
Lesion size	≤5 cm	4	0.48 (0.28−0.68)	0.52	0.91 (0.84−0.98)	0.18
	NR	15	0.57 (0.46−0.67)		0.85 (0.80−0.91)	
No. of tumors (n)	≥100	14	0.56 (0.45−0.66)	0.97	0.86 (0.80−0.91)	0.01
	<100	5	0.52 (0.33−0.72)		0.89 (0.81−0.97)	
Lesions	S	7	0.54 (0.39−0.69)	0.71	0.85 (0.77−0.93)	0.00
	Others	12	0.55 (0.43−0.67)		0.88 (0.82−0.93)	
IBIS (days)	≤30	15	0.58 (0.48−0.68)	0.28	0.84 (0.79−0.90)	0.00
	>30	4	0.41 (0.21−0.61)		0.93 (0.88−0.99)	
Macro VI	N	13	0.56 (0.45−0.67)	0.97	0.88 (0.82−0.93)	0.05
	U	6	0.52 (0.35−0.69)		0.85 (0.75−0.94)	
BR	Y	13	0.51 (0.40−0.62)	0.17	0.90 (0.87−0.94)	0.26
	U	6	0.63 (0.49−0.78)		0.76 (0.66−0.86)	
BI	Y	2	0.52 (0.24−0.81)	0.81	0.77 (0.56−0.97)	0.05
	U	17	0.55 (0.45−0.65)		0.88 (0.83−0.92)	

Data in parentheses are 95% confidence interval (CI). MFS, magnetic field strength; IBIS, interval between imaging and surgery; Macro VI, macrovascular invasion; BR, blindness to reference; BI, blindness to index test; NR, no restrictions; S, single; M, multiple; U, unclear; N, no; Y, yes.

### 3.6 Sensitivity Analysis and Publication Bias

**Figure 7:**
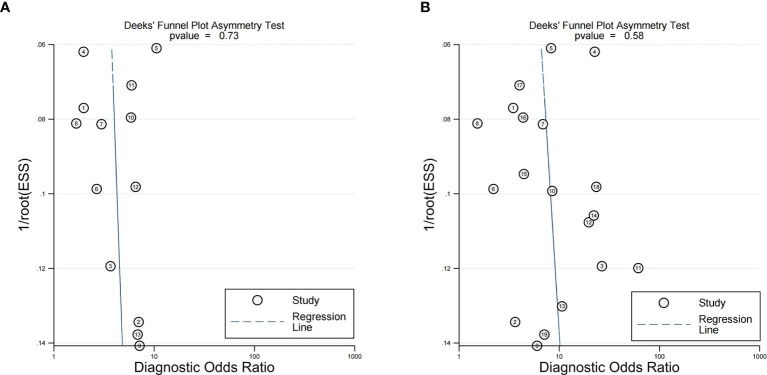
Deeks' functional plots of peritumoral enhancement on the arterial phase **(A)** and peritumoral hypointension on the hepatobiliary phase **(B)**.

The results of sensitivity analysis, performed for the two imaging features by eliminating included articles one by one, revealed that none of the articles had any significant effect on the pooled results. There was no significant publication bias in Deeks’ funnel plot asymmetry test of peritumoral enhancement on AP (*P* = 0.73) (**Figure 7A**) and peritumoral hypointensity on HBP (*P* = 0.58) (**Figure 7B**).

## 4 Discussion

MVI is a risk factor for HCC recurrence, and the preoperative noninvasive prediction of MVI remains challenging. In our meta-analysis, based on peritumoral imaging findings, the results revealed that both peritumoral enhancement on AP and peritumoral hypointensity on HBP had high SPE but low SEN, which indicated that Gd-EOB-DTPA-enhanced MRI is helpful as a noninvasive, excluded diagnosis for predicting MVI in HCC preoperatively.

The relationship between peritumoral enhancement and the presence of MVI could be understood as that corona enhancement is a hemodynamic perfusion change due to disturbed portal venous drainage (40–42). Furthermore, the reasons why the peritumoral signal was low during HBP could be explained as follows: the occlusion of the intrahepatic portal vein and insufficient compensation of the hepatic arterial flow lead to hepatic parenchyma injury, edema, hepatocyte depletion, and fibrosis (43). Moreover, previous articles have confirmed a positive correlation between the enhancement ratio of HCCs in the HBP of Primovist-enhanced MRI and the expression of organic anion–transporting polypeptide (OATPs) and multidrug-resistant proteins (MRPs); of note, gadoxetic acid disodium is absorbed by OATP8 and excreted by MRP3 (44, 45). Additionally, tumor invasion into small portal vein branches probably leads to hemodynamic perfusion changes and then affects the expression of OATP8 and MRP3 in hepatocytes, which may have an impact on hepatic function and decrease gadoxetic acid uptake into hepatocytes near tumors, leading to peritumoral hypointensity on HBP (19, 23).

The preoperative imaging of peritumoral tissue showing MVI has been applied to conventional CT and MRI. However, Chou CT et al. found that peritumoral enhancement on CT was not a significant risk factor for MVI (46). Chun Yang et al. also claimed that peritumoral enhancement did not show a statistically significant association with MVI (*P* > 0.05), when performing MRI scans using non-hepatocyte-specific contrast agents, called Magnevist (47). However, in our study, peritumoral enhancement on Gd-EOB-DTPA-enhanced MRI had an association with MVI and had a high SPE of 87%. This may be related to the imaging principles of CT and non-hepatocyte-specific contrast agents. Moreover, since the drainage of contrast from the tumor vein to the peritumoral parenchymal sinusoids and portal venules is an extremely transient process, it inevitably causes transient and severe respiratory motion. In addition, respiratory motion artifacts affect all dynamic phases, especially during the arterial phase (48). Additionally, Wybranski C et al. suggested that Gd-EOB-DTPA-related respiratory motion could not be improved by a series of standard pre-scan patient preparations including breath-holding training (48). This might be the reason why peritumoral enhancement had a low SEN. As a result, Kim H et al. proposed that a more accurate assessment of peritumoral enhancement should be done by a multi-arterial phase study (49). Although an SEN of 50% of peritumoral enhancement in the present study is low, it has been greatly improved compared with a previous meta-analysis that included traditional CT (a pooled SEN of 0.29) (16). It is undeniable that Gd-EOB-DTPA-enhanced MRI has some advantages in detecting MVI. However, peritumoral enhancement is more often seen in hypervascular progressed HCC. While peritumoral enhancement was not present in many hypovascular HCCs, it is reported that the double hypointensity in the portal/venous and HBP were highly suggestive of hypovascular HCC. However, the diagnostic performance of double hypointensity for MVI has not been reported; therefore, it needs further investigation in the future (50).

There were few studies performed to detect MVI utilizing the peritumoral tissue imaging performance of Gd-EOB-DTPA-enhanced MRI. However, a study conducted by Ahn SY et al. (18) found no significant correlation between peritumoral hypointensity on HBP and MVI (*P* > 0.05). These authors explained that peritumoral hypointensity was not a common observation (it was found in 25% of HCCs) and attributed this discrepancy to the differences in patient populations, small sample size, and low SEN (38.3%) in the research of Kim KA et al. (19). However, in present study, the SEN and SPE of peritumoral hypointensity were 0.55 and 0.87, respectively, which had some clinical applicability, especially as an exclusionary diagnostic tool. Further, Kim KA et al. (19) suggested that the SEN of detecting MVI with peritumoral hypointensity is relatively low, which may be due to the fact that the prevalence of MVI in certain tumors is not associated with any changes in peritumoral hepatocyte function. We hypothesize that some tumor functional changes occur later. This could also be the cause for the low SEN of peritumoral hypointensity in the current study.

Furthermore, in a high-quality study using Gd-BOPTA-enhanced MRI, the SEN and SPE of peritumoral enhancement on AP and peritumoral hypointensity on HBP were 0.23 and 0.95, respectively, and 0.49 and 0.89, respectively (51). Overall, the gap of peritumoral hypointensity between the study results and the present study was not significant, but the difference in peritumoral enhancement was a little higher. In particular, the results showed that the missed diagnosis rate using Gd-EOB-DTPA was relatively lower compared to Gd-BOPTA, but it needs to be verified by multicenter and large sample studies in the future.

According to our meta-analysis, both peritumoral enhancement and peritumoral hypointensity are key factors in predicting MVI and demonstrate moderate accuracy, which are consistent with the findings of most previous studies (8, 19, 21, 25–29, 32, 33). Different imaging techniques to explore the relationship between MVI with peritumoral imaging all showed a low SEN. In the future, how to improve its SEN will be a new research direction. For example, we speculate on whether the radiomics of peritumoral imaging or quantitative analysis to determine MVI can further improve its accuracy. As shown in the Huang M et al. study, peritumoral enhancement and peritumoral hypointense do not always coexist and a more accurate prediction model for MVI is needed (8). Therefore, whether a model with a combination of multiple imaging presentations has a higher clinical application deserves further investigation.

Assessing accuracy is necessarily preceded by assessing heterogeneity. In our study, ten covariates and seven covariates were found to be significant sources of heterogeneity for peritumoral enhancement and peritumoral hypointensity on HBP, respectively. It indicated that the future articles should pay attention to the basic information of the included patients, including the region, mean age, and number and size of included lesions, and should not ignore the interval between imaging and surgery and blindness to reference tests, so as to improve the quality of research.

In this investigation, there are several flaws. First, the population included in those studies was predominantly Asian, which meant that it was not possible to exclude out the potentiality of selection bias. Second, image interpretation depends on the observer so that subjectivity is inevitable to some extent. Finally, the data provided were inadequate for further investigation on peritumoral imaging findings. Therefore, larger multicenter studies are required for a more accurate assessment of the ability of Gd-EOB-DTPA-enhanced MRI to predict MVI.

In conclusion, our study found that the results of peritumoral enhancement on AP and peritumoral hypointensity on HBP showed high SPE but low SEN. This indicates that the peritumoral imaging features on Gd-EOB-DTPA-enhanced MRI can be used as a noninvasive, excluded diagnosis for predicting hepatic MVI in HCC preoperatively. Moreover, the results of this analysis should be updated when additional data become available. In the future, how to improve its SEN will be a new research direction.

## Data Availability Statement

The original contributions presented in the study are included in the article/**Supplementary Material**. Further inquiries can be directed to the corresponding author.

## Author Contributions

YW and LY proposed the concepts and designed the study. YW wrote the manuscript. MZ was responsible for the study selection and data extraction. YL and XC contributed to analyze the statistics. GZ and LY revised this manuscript. All authors read and approved the final manuscript.

## Funding

This study was supported by the Diagnosis and Treatment Projects of Major Digestive System Diseases, the Department of Science and Technology of Sichuan Province (No. 2021YFS0375).

## Conflict of Interest

The authors declare that the research was conducted in the absence of any commercial or financial relationships that could be construed as a potential conflict of interest.

## Publisher’s Note

All claims expressed in this article are solely those of the authors and do not necessarily represent those of their affiliated organizations, or those of the publisher, the editors and the reviewers. Any product that may be evaluated in this article, or claim that may be made by its manufacturer, is not guaranteed or endorsed by the publisher.
