# Supplementary material for: Peritumoral Imaging Manifestations on Gd-EOB-DTPA-Enhanced MRI for Preoperative Prediction of Microvascular Invasion in Hepatocellular Carcinoma: A Systematic Review and Meta-Analysis
Source: Front Oncol. 2022 Jun 24;12:907076. doi: 10.3389/fonc.2022.907076 (PMC9263828; doi:10.3389/fonc.2022.907076)
Supplement: Supplementary file 1 [file DataSheet_1.docx]

**Literature search strategy:**

**Up until Feb 24, 2022：**

**PubMed database：**

①(Carcinoma, Hepatocellular[Mesh]) OR (((((((((((((((((((Carcinomas, Hepatocellular[Title/Abstract])) OR (Hepatocellular Carcinomas[Title/Abstract])) OR (Liver Cell Carcinoma, Adult[Title/Abstract])) OR (Liver Cancer, Adult[Title/Abstract])) OR (Adult Liver Cancer[Title/Abstract])) OR (Adult Liver Cancers[Title/Abstract])) OR (Cancer, Adult Liver[Title/Abstract])) OR (Cancers, Adult Liver[Title/Abstract])) OR (Liver Cancers, Adult[Title/Abstract])) OR (Liver Cell Carcinoma[Title/Abstract])) OR (Carcinoma, Liver Cell[Title/Abstract])) OR (Carcinomas, Liver Cell[Title/Abstract])) OR (Cell Carcinoma, Liver[Title/Abstract])) OR (Cell Carcinomas, Liver[Title/Abstract])) OR (Liver Cell Carcinomas[Title/Abstract])) OR (Hepatocellular Carcinoma[Title/Abstract])) OR (Hepatoma[Title/Abstract])) OR (Hepatomas[Title/Abstract]))

② ((invasion[Title/Abstract])) AND ((((microvascular[Title/Abstract])) OR (microvessel[Title/Abstract])) OR (microscopic[Title/Abstract]))

③(("Magnetic Resonance Imaging"[Mesh]) OR (((((((((((((((((((((((((((((((((((((((((((Imaging, Magnetic Resonance[Title/Abstract]) OR (NMR Imaging[Title/Abstract])) OR (Imaging, NMR[Title/Abstract])) OR (Tomography, NMR[Title/Abstract])) OR (Tomography, MR[Title/Abstract])) OR (MR Tomography[Title/Abstract])) OR (NMR Tomography[Title/Abstract])) OR (Steady-State Free Precession MRI[Title/Abstract])) OR (Steady State Free Precession MRI[Title/Abstract])) OR (Zeugmatography[Title/Abstract])) OR (Imaging, Chemical Shift[Title/Abstract])) OR (Chemical Shift Imagings[Title/Abstract])) OR (Imagings, Chemical Shift[Title/Abstract])) OR (Shift Imaging, Chemical[Title/Abstract])) OR (Shift Imagings, Chemical[Title/Abstract])) OR (Chemical Shift Imaging[Title/Abstract])) OR (Tomography, Proton Spin[Title/Abstract])) OR (Proton Spin Tomography[Title/Abstract])) OR (Magnetization Transfer Contrast Imaging[Title/Abstract])) OR (MRI Scans[Title/Abstract])) OR (MRI Scan[Title/Abstract])) OR (Scan, MRI[Title/Abstract])) OR (Scans, MRI[Title/Abstract])) OR (fMRI[Title/Abstract])) OR (MRI, Functional[Title/Abstract])) OR (Functional MRI[Title/Abstract])) OR (Functional MRIs[Title/Abstract])) OR (MRIs, Functional[Title/Abstract])) OR (Functional Magnetic Resonance Imaging[Title/Abstract])) OR (Magnetic Resonance Imaging, Functional[Title/Abstract])) OR (Spin Echo Imaging[Title/Abstract])) OR (Echo Imaging, Spin[Title/Abstract])) OR (Echo Imagings, Spin[Title/Abstract])) OR (Imaging, Spin Echo[Title/Abstract])) OR (Imagings, Spin Echo[Title/Abstract])) OR (Spin Echo Imagings[Title/Abstract]))

④ (gadolinium ethoxybenzyl DTPA [Mesh]) OR ((((((((((Gd-EOB-DTPA[Title/Abstract])) OR (gadolinium ethoxybenzyl diethylenetriaminepentaacetic acid[Title/Abstract])) OR (gadoxetic acid[Title/Abstract])) OR (gadoxetic acid disodium[Title/Abstract])) OR (gadoxetate disodium[Title/Abstract])) OR (disodium gadoxetate[Title/Abstract])) OR (gadolinium (4S)-4-(4-ethoxybenzyl)-3,6,9-tris(carboxylatomethyl)-3,6,9-triazaundecanoic acid disodium salt[Title/Abstract])) OR (Eovist[Title/Abstract])) OR (Primovist[Title/Abstract]))

⑤ = ③ AND ④

Final search strategy: ① AND ② AND ⑤ ;

Final record: 94 studies.

**Embase database：**

①'liver cell carcinoma'/exp OR 'carcinoma, hepatocellular':ab,ti OR ‘Carcinomas, Hepatocellular':ab,ti OR ‘Hepatocellular Carcinomas':ab,ti OR ’Liver Cell Carcinoma, Adult':ab,ti OR ’Liver Cancer, Adult':ab,ti OR ’Adult Liver Cancer':ab,ti OR ’Adult Liver Cancers':ab,ti OR ’Cancer, Adult Liver':ab,ti OR ’Cancers, Adult Liver':ab,ti OR ’Liver Cancers, Adult':ab,ti OR ’Liver Cell Carcinoma':ab,ti OR ’Carcinoma, Liver Cell':ab,ti OR ’Carcinomas, Liver Cell':ab,ti OR ’Cell Carcinoma, Liver':ab,ti OR ’Cell Carcinomas, Liver':ab,ti OR ’Liver Cell Carcinomas':ab,ti OR ’Hepatocellular Carcinoma':ab,ti OR ’Hepatoma':ab,ti OR ’Hepatomas':ab,ti

②’microvascular':ab,ti OR ’microvessel':ab,ti OR ’microscopic':ab,ti

③’invasion':ab,ti

④=③ AND ②

⑤'nuclear magnetic resonance imaging'/exp OR ’Magnetic Resonance Imaging':ab,ti OR ’Imaging, Magnetic Resonance':ab,ti OR ’NMR Imaging':ab,ti OR ’Imaging, NMR':ab,ti OR ’Tomography, NMR':ab,ti OR ’Tomography, MR':ab,ti OR ’MR Tomography':ab,ti OR ’NMR Tomography':ab,ti OR ’Steady-State Free Precession MRI':ab,ti OR ’Steady State Free Precession MRI':ab,ti OR ’Zeugmatography':ab,ti OR ’Imaging, Chemical Shift':ab,ti OR ’Chemical Shift Imagings':ab,ti OR ’Imagings, Chemical Shift':ab,ti OR ’Shift Imaging, Chemical':ab,ti OR ’Shift Imagings, Chemical':ab,ti OR ’Chemical Shift Imaging':ab,ti OR ’Tomography, Proton Spin':ab,ti OR ’Proton Spin Tomography':ab,ti OR ’Magnetization Transfer Contrast Imaging':ab,ti OR ’MRI Scans':ab,ti OR ’MRI Scan':ab,ti OR ’Scan, MRI':ab,ti OR ’Scans, MRI':ab,ti OR ’fMRI':ab,ti OR ’MRI, Functional':ab,ti OR ’Functional MRI':ab,ti OR ’Functional MRIs':ab,ti OR ’MRIs, Functional':ab,ti OR ’Functional Magnetic Resonance Imaging':ab,ti OR ’Magnetic Resonance Imaging, Functional':ab,ti OR ’Spin Echo Imaging':ab,ti OR ’Echo Imaging, Spin':ab,ti OR ’Echo Imagings, Spin':ab,ti OR ’Imaging, Spin Echo':ab,ti OR ’Imagings, Spin Echo':ab,ti OR ’Spin Echo Imagings':ab,ti

⑥'gadoxetic acid'/exp OR ’gadolinium ethoxybenzyl DTPA':ab,ti OR ’Gd-EOB-DTPA':ab,ti OR ’gadolinium ethoxybenzyl diethylenetriaminepentaacetic acid':ab,ti OR ’gadoxetic acid disodium':ab,ti OR ’gadoxetate disodium':ab,ti OR ’disodium gadoxetate':ab,ti OR ’gadolinium (4S)-4-(4-ethoxybenzyl)-3,6,9-tris(carboxylatomethyl)-3,6,9-triazaundecanoic acid disodium salt':ab,ti OR ’Eovist':ab,ti OR ’Primovist':ab,ti

⑦=⑤ AND ⑥

Final search strategy:① AND ④ AND ⑦ ;

Final record: 72 studies.

**Cochrane library database：**

#1 MeSH descriptor: [Carcinoma, Hepatocellular] explode all trees

#2 carcinoma, hepatocellular OR Carcinomas, Hepatocellular OR Hepatocellular Carcinomas OR Liver Cell Carcinoma, Adult OR Liver Cancer, Adult OR Adult Liver Cancer OR Adult Liver Cancers OR Cancer, Adult Liver OR Cancers, Adult Liver OR Liver Cancers, Adult OR Liver Cell Carcinoma OR Carcinoma, Liver Cell OR Carcinomas, Liver Cell OR Cell Carcinoma, Liver OR Cell Carcinomas, Liver OR Liver Cell Carcinomas OR Hepatocellular Carcinoma OR Hepatoma OR Hepatomas:ti,ab,kw

#3= #1 or #2

#4 microvascular OR microvessel OR microscopic:ti,ab,kw

#5 invasion:ti,ab,kw

#6= #4 AND #5

#7 MeSH descriptor: [Magnetic Resonance Imaging] explode all trees

#8 Imaging, Magnetic Resonance OR NMR Imaging OR Imaging, NMR OR Tomography, NMR OR Tomography, MR OR MR Tomography OR NMR Tomography OR Steady-State Free Precession MRI OR Steady State Free Precession MRI OR Zeugmatography OR Imaging, Chemical Shift OR Chemical Shift Imagings OR Imagings, Chemical Shift OR Shift Imaging, Chemical OR Shift Imagings, Chemical OR Chemical Shift Imaging OR Tomography, Proton Spin OR Proton Spin Tomography OR Magnetization Transfer Contrast Imaging OR MRI Scans OR MRI Scan OR Scan, MRI OR Scans, MRI OR fMRI OR MRI, Functional OR Functional MRI OR Functional MRIs OR MRIs, Functional OR Functional Magnetic Resonance Imaging OR Magnetic Resonance Imaging, Functional OR Spin Echo Imaging OR Echo Imaging, Spin OR Echo Imagings, Spin OR Imaging, Spin Echo OR Imagings, Spin Echo OR Spin Echo Imagings:ti,ab,kw

#9=#7 OR #8

#10 MeSH descriptor: [Gadolinium DTPA] explode all trees

#11 gadolinium ethoxybenzyl DTPA OR Gd-EOB-DTPA OR gadolinium ethoxybenzyl diethylenetriaminepentaacetic acid OR gadoxetic acid OR gadoxetic acid disodium OR gadoxetate disodium OR disodium gadoxetate OR Eovist OR Primovist:ti,ab,kw

#12= #10 OR #11

#13= #9 AND #12

Final search strategy: #14 = #3 AND #6 AND #13

Final record: 2 studies.
